# Mixed pneumonic plague and nosocomial MDR-bacterial infection of lung: a rare case report

**DOI:** 10.1186/s12890-018-0656-y

**Published:** 2018-05-29

**Authors:** Voahangy Andrianaivoarimanana, Eric Bertherat, Rojo Rajaonarison, Tiana Rakotondramaro, Christophe Rogier, Minoarisoa Rajerison

**Affiliations:** 1 Plague Unit- Institut Pasteur de Madagascar, BP1274 Ambatofotsikely, 101 Antananarivo, Madagascar; 2 Department of Infectious Hazard Management, World Health Organization, Avenue Appia 20, CH-1211, 27 Geneva, Switzerland; 3 Centre Hospitalier Anti-pesteux d’Ambohimiandra, 101 Antananarivo, Madagascar; 4 Centre Hospitalier de District de Miarinarivo, Antananarivo, Madagascar; 5 Institut Pasteur de Madagascar, BP1274 Ambatofotsikely, 101 Antananarivo, Madagascar; 6 Present address: Service de Santé des Armées, Direction Centrale, Division Expertise et Stratégie Santé de Défense, 60 Boulevard du Général Martial Valin – CS21623, 75509 Paris Cedex 15, France

**Keywords:** *Yersinia pestis*, *Stenotrophomonas maltophilia*, Pneumonia, Madagascar

## Abstract

**Background:**

Plague is a life-threatening disease caused by the bacterium, *Yersinia pestis*. Madagascar is the leading country for human plague cases worldwide. Human plague is a serious disease, particularly in its septicaemic and pneumonic forms. We report a case of pneumonic plague co-infected by a MDR-*Stenotrophomonas maltophilia.*

**Case presentation:**

A 24 year-old man originated from Soavinandriana, a plague focus, felt uneasy and developed high fever with chills. He started treatment by himself, by private medical care and by a traditional healer for nine days moving several times from place to place. His condition had deteriorated when he presented to a district hospital with a syndrome of dyspnea, bronchial rale and altered state of consciousness. Two days later, plague diagnosis, performed as a last resort, revealed a positive F1 antigen on rapid diagnostic test. Additional tests (pla PCR and plague serology) evidenced a *Y. pestis* infection. However, streptomycin treatment did not achieve a complete recovery as the course of disease was complicated by the presence of MDR-*S. maltophilia* in his lung. This opportunistic infection could have been favored by an immunosuppression due to *Y. pestis* pulmonary infection and probably been acquired during his stay at a District Hospital. He was treated with a combination of ciprofloxacin and gentamycin and recovered fully.

**Conclusions:**

Pneumonic plague infection may promote another virulent or avirulent bacterial infection particularly when it is not initially suspected. However, coinfection is rarely described and its occurrence frequency is unknown. In middle or low resources areas, which is the case of most plague endemic countries, control and prevention of infections in health facilities is not optimal. Co-infection with an opportunistic pathogen agent, such as *S. maltophilia,* is a risk which must not be disregarded as demonstrated by this case report. When deciding of a national control strategy, it should be taken into account in the choice of the first line treatment.

## Background

Plague is a rapidly progressing disease in humans caused by the bacterium, *Yersinia pestis* [1]. Transmission of bubonic plague to human occurs after the bite of an infectious flea. Less frequently, infection may also occur by inhalation of infectious respiratory droplets spread from a patient suffering from pneumonic plague. In that case, the fatality rate is almost 100% if the patient is left untreated. Pneumonic plague severity is due to *Y. pestis* ability to evade the host immune system and also to the rapid establishment, by *Y. pestis* infection, of a permissive environment for microbial proliferation in the lung [2]. According to the World Health Organization (WHO), 3248 cases of human plague have been reported between 2010 and 2015 by 11 countries from Africa, the Americas and Asia. During this period, Madagascar accounted for 74% of all cases reported worldwide with 18% of case fatality rate, making this island the most affected country in the world [3]. Since its introduction in Madagascar in 1898, plague is endemic within a large area in the central and in the northern highlands above 800 m in elevation [4]. Although plague is mainly a rural disease, some sporadic cases may occur particularly in the urban setting [5]. In Madagascar, methods for plague diagnosis includes F1 antigen RDT, serology, bacteriological culture on selective media at 26–28 °C, and *Y. pestis* identification by biochemical profile using API20E kit and by bacteriophage lysis test [3]. Antibiotics susceptibility of each isolated strain is surveyed. Molecular biology is not used routinely. As per the Plague National Control Program (PNCP), specific treatment of pneumonic plague requires streptomycin injection during 8 days. However, this therapeutic strategy may fail in presence of MDR- *Y. pestis* [6] or mixed infection involving other MDR-pathogen. Here, we describe the features and medical history of a case of pneumonic plague complicated by a MDR-*Stenotrophomonas maltophilia* co-infection in a patient who travelled in different locations before reaching the capital Antananarivo as well as the case-management. *S. maltophilia* is an aerobic gram-negative bacillus which is an opportunistic and nosocomial pathogen causing blood-stream infections and pneumonia with considerable morbidity in immunocompromised patients [7, 8].

## Case presentation

**Fig. 1:**
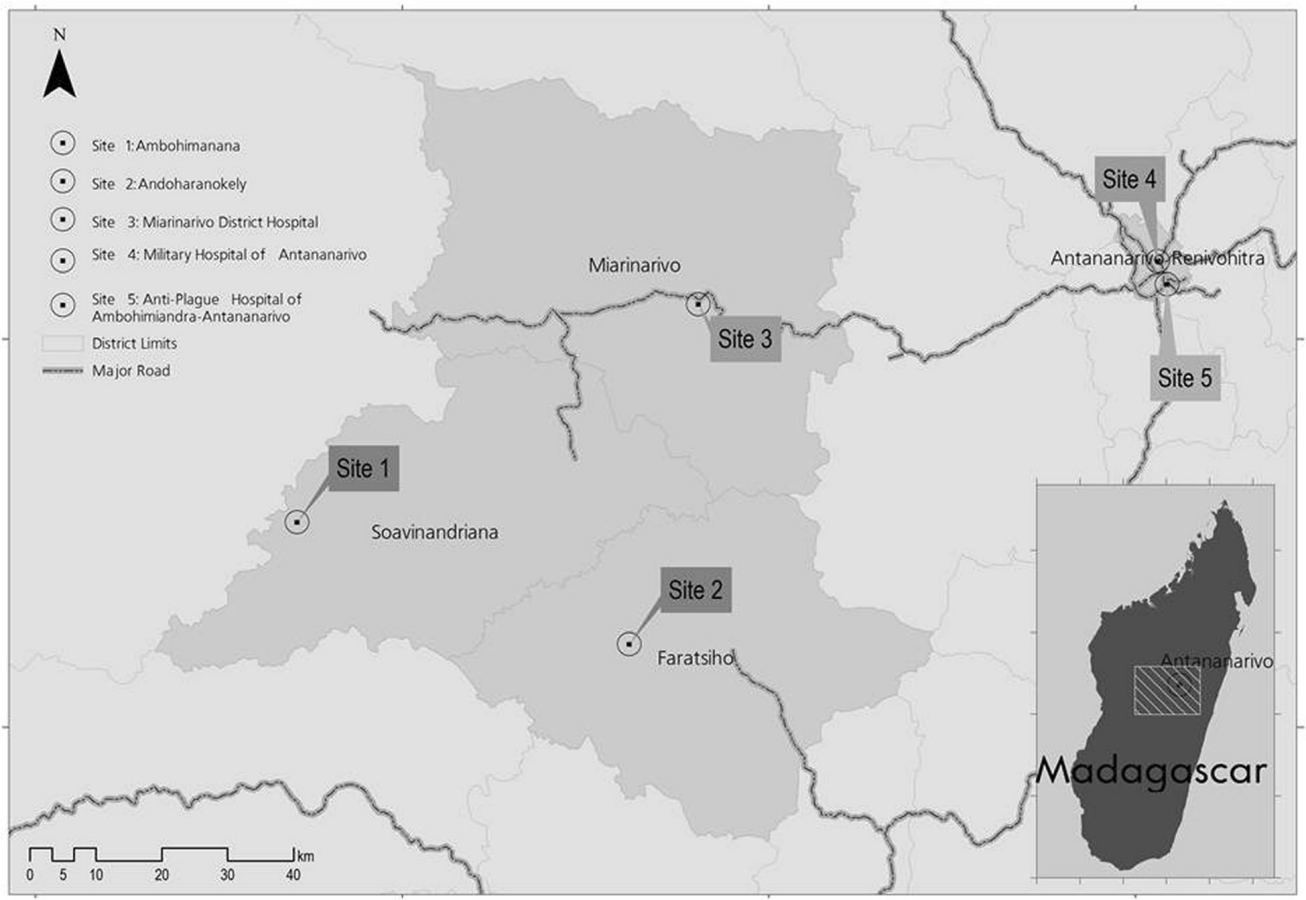
Map emphasizing on the patient’s itinerary. Site 1 Ambohimanana-Soavinandriana: patient’s village; Site 2 Andoharanokely-Faratsiho: Traditional healer’s village; Site 3 Miarinarivo District Hospital; Site 4 Military Hospital of Antananarivo; Site 5 Anti-Plague Hospital of Ambohimiandra-Antananarivo

**Fig. 2:**
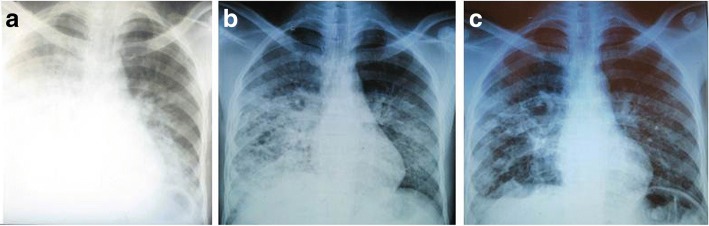
Chest X-ray of the pneumonic plague patient. **a** on admission at Miarinarivo District Hospital: chest X-ray image revealed right pleural effusion and radio-opacities of left lower lobe, (**b**) On presentation to the Military Hospital of Antananarivo (10 days after A): enlargement of left opacities and appearance of right nodular opacity, decrease of right pleural effusion, and (**c**) Upon discharge at Anti-plague Hospital of Ambohimiandra- Antananarivo (9 days after B), decrease of right opacity and left pleural effusion

On December 30, 2013, a 24 year-old man developed high fever with chills and took self-medication of trimethoprim-sulfamethoxazole (2x80mg-2x400mg respectively) and antipyretic [9]. Later the same day, he sought private medical care and received antimalarial treatment (3 quinine injections). Two days earlier, he transported on his motorcycle a sick person who died later for unknown reasons. He lived in the village of Ambohimanana within the District of Soavinandriana (Fig. 1, Site 1), one of the plague foci of the central highlands of Madagascar. In the absence of improvement, he visited a traditional healer at Andoharanokely- Faratsiho (Fig. 1, Site 2) on December 31, and received herbal medicines and massage for 5 days. His condition continued to decline with severe asthenia and fever. The traditional healer authorized him to get a treatment by modern medicine. On January 7, 2014, an agent of a first-level health center (Fig. 1, Site 2) gave him primary available treatment (betalactam, antipyretic, bronchodilator and a prokinetic agent) and evacuated him to Miarinarivo District Hospital (MDH) (Fig. 1, Site 3) for a syndrome of dyspnea, bronchial rale and altered state of consciousness. On his admission to MDH on January 8, 2014, he had a temperature of 39.5 °C and a heart rate of 120 b/min. The chest radiograph showed right pleural effusion and left radio-opacities of the lower lobe (Fig. 2a). Rapid diagnostic test (RDT) for malaria and Widal-Felix serology for typhoid fever were both negative. Acid-fast bacilli from two sputa cultures were also negative. Therefore, he was treated for pneumonia with ceftriaxone (1 g) intravenously (IV).

Considering the fact that he was living in a known plague region, RDT for *Y. pestis* F1 antigen [10] was performed on sputum as a differential diagnosis the next day and was markedly positive, suggesting a pneumonic plague infection. Intramuscular streptomycin treatment was immediately initiated after the diagnosis according to the PNCP of the Malagasy Ministry of Health: 0,5 g every 3 h for 2 days followed by 0,5 g every 4 h for 2 days and 1 g twice a day for 4 days. This sputum sample was sent to the Central Laboratory for Plague at Institut Pasteur de Madagascar for bacteriological confirmation [11] but did not yield *Y. pestis* strain; instead *Citrobacter freundii* was identified. After completing an 8-day course of streptomycin treatment, the patient’s condition remained stable without signs of recovery. Therefore, on January 17, 2014, he was discharged from the hospital by his family against medical advice and sought treatment at the Military Hospital of Antananarivo (MHA) (Fig. 1, Site 4). On his admission at MHA on January 18, 2014 at 11:30 a.m., a second chest radiograph revealed an enlargement of left opacities and appearance of right nodular opacity, but a decrease of right pleural effusion (Fig. 2b). His status has still altered and marked with asthenia, anorexia, weight loss, greyish complexion, colored conjunctiva with a temperature of 38.5 °C. During physical examination, he had a cough with purulent sputum associated with a mean abundance of hemoptysis, dyspnea, bilateral crackles and breath-sounds rales. The examination of lymph nodes was normal. Biological analysis showed a rise in the level of C-reactive protein (12 mg/ml) revealing an inflammatory syndrome. In addition, the second sputum sample collected remained positive for *Y. pestis* F1 antigen. In order to limit exposure to the disease, the patient was transferred to the Anti-Plague Hospital of Ambohimiandra-Antananarivo (APHAA) (Fig. 1, Site 5) the same day at 01:00 p.m. Once admitted, he was isolated in an individual room. Given the positive result of *Y. pestis* F1 antigen and the absence of patient’s health improvement, another therapeutic scheme was initiated and consisted of a combination of IV ciprofloxacin (400 to 600 mg twice per day for 6 days) and IV gentamycin (3 mg/kg per day, for 6 days). Bacteriological culture of the second sputum sample also failed to isolate *Y. pestis*, however growing colonies on cefsulodin-irgasan-novobiocin medium were identified as *S. maltophilia* on API20E biochemical strip. Antibiotic susceptibility test using a disk diffusion method as per the Clinical and Laboratory Standards Institute guidelines showed that *S. maltophilia* isolate was sensitive to trimethoprim-sulfamethoxazole, ciprofloxacin and chloramphenicol, but was resistant to tetracycline, streptomycin, gentamycin and ampicillin. Strain *Escherichia coli* ATCC®25,922 was used as control. Moreover, plague ELISA serology testing in paired serum samples (Ethical Committee authorization 068- MSANPE of July 29, 2011) performed at a 7-day interval showed a three-fold rise in anti-F1 immunoglobulin G titer [12] and PCR test targeting *Y. pestis* plasminogen activator (pla) gene [13] was positive on all sputum samples.

The suggested bi-therapy treatment led to the patient’s condition improvement and recovery. Upon discharge, on January 27, 2014, his third chest X-ray demonstrated a decrease of right opacity and left pleural effusion (Fig. 2c).

## Discussion

Madagascar is one of the most active plague regions in the world and was the origin of a natural multi-drug resistant strain of *Y. pestis* [6]. Incidence has declined in the last 2 years but there has been an increase in the overall case-fatality rate (23% in 2015) associated with the high frequency of pneumonic form (23.3%) [3]. Within 24–36 h after the onset of symptoms, pneumonic plague progresses to an irreversible and lethal syndrome that cannot be effectively treated [14]. Indeed, most pneumonic plague patients succumb in less than 3 days without prompt and adequate treatment. For the present case, which is most likely a primary pneumonic infection, the early doses of trimethoprime-sulfamethoxazole self-administered might have induced a latent phase in pneumonic plague infection, a delayed infection course which allowed the patient to survive despite a delay of plague treatment. It was demonstrated that the most common indications for self-medication were pain, fever and cough [9]. In addition, antibiotic medication rapidly clears the sputum of plague bacilli, so that a patient is generally not infective within hours after initiation of effective antibiotic treatment [15]. In this report, clinical presentation and epidemiological evidence (residence in a plague endemic area) are consistent with a pneumonic plague infection. Additionally, laboratory results met the criterion of the WHO standard case definition for a “Confirmed plague case” [5]. It is noteworthy that F1 antigen is specific to *Y. pestis* [16]*,* temperature-stable and is excreted in large amount in samples from plague infected people [10]. The failure of *Y. pestis* isolation by bacterial culture is not uncommon if antibiotic treatment was administered before sampling [17, 18]. Positive results of F1 antigen RDT on all sputum samples showed that *Y. pestis* F1 antigen could persist and be detected several days after the beginning of treatment, when the plague bacillus is no longer viable [10, 17, 19]; for this case, F1 antigen persisted for 10 days after the beginning of treatment and 20 days after disease onset. Due to the failure of the culture, sensitivity of *Y. pestis* to streptomycin was not tested; however a resistance is doubtful because the patient would have likely succumbed to pneumonic plague infection if the complete streptomycin treatment was not effective. The isolation of MDR-*S. maltophilia* from his sputum collected at MDH suggested that the patient had a mixed infection with *Y. pestis* and *S. maltophilia.* This nosocomial infection has probably been acquired during his stay at this hospital and may have been favored by patient immunocompromised condition. Without other medical information, his immune response maybe attenuated by the course of his disease or by malnutrition with a notable weight loss. However, it has been shown that pulmonary infection by *Y. pestis* may establish a permissive environment for proliferation of usually nonpathogenic bacteria [2]. In middle or low resources areas, which is the case of most plague endemic countries, control and prevention of infections in health facilities is not optimal. Co-infection with an opportunistic pathogen agent is a risk which must not be disregarded. Indeed, *S. maltophilia* is an important emergent opportunistic pathogen, causing lung colonization or pulmonary infections among immunosuppressed patients. According to available clinical information, distinction between *S. maltophilia* colonization and infection can be confusing [20]. His chest X-ray, 10 days after MDH admission, showed right nodular opacity (Fig. 2b) and might be evocative of *S. maltophilia* colonization or infection of a severely impaired host [21, 22].

*S. maltophilia* infection/colonization management is often problematic due to its inherent resistance to multiple broad-spectrum antibiotic agents [21]. Thus, for the presenting case, the initial streptomycin treatment was effective for *Y. pestis* infection but not for *S. maltophilia*. The use of ciprofloxacin in secondary bi-therapy allowed the recovery of the patient and this antibiotic is also recommended as an alternative treatment for *S. maltophilia* infection [23]. Indeed, recent study also supported the broader use of oral ciprofloxacin for treatment of human plague including pneumonic form. Its bactericidal activity is comparable to that of streptomycin, and its mode of administration is less restrictive; more acceptable for exposed population [24]. In addition, a wide range of antibiotics was already in vitro tested for their susceptibility to geographically diverse strains of *Y. pestis* which provide alternatives for plague treatment [25]. Another study had also investigated the susceptibility patterns of *Y. pestis* strains from diverse sources to 12 antimicrobial agents and showed that a large panel of *Y. pestis* remained susceptible not only to drugs traditionally used to treat plague (streptomycin, doxycycline and chloramphenicol), but also to newer agents (broad-spectrum cephalosporins and quinolones). The most active compounds against *Y. pestis* included ceftriaxone and trimethoprim-sulfamethoxazole, quinolones [26].

The severe pneumonic plague outbreak which occurred in urban context in Madagascar recently [27] questioned the pertinence of maintaining streptomycin as the first line treatment. The challenge of the clinical diagnosis in the context of an urban outbreak associated to the intense circulation of other potential causes of pneumonia could justify the choice of an antibiotic with a broader spectrum. This case report suggests that the possibility of mixed infection, especially in a nosocomial context, must be part of this strategic discussion.

To date, coinfection involving pneumonic plague and other pulmonary infections has been rarely described. Indeed, Quan et al. reported the recovery of *Y. pestis* and other respiratory pathogens from mammals and ectoparasites but not in human during plague investigations [28]. However in 2004, two cases of confirmed infection for plague and leptospirosis have been observed in the Democratic Republic of Congo, both of which can cause severe pulmonary manifestations [29]. To our knowledge, this study is the first describing a co-infection with *Y. pestis* and *S. maltophilia* in Madagascar and emphasizes on the complicated management of such pneumonic infections.

## Conclusion

In conclusion, although mixed infection of lung with *Y. pestis* and *S. maltophilia* is rare, a combined therapy should be discussed when the condition of a plague patient does not improve under an appropriate treatment with streptomycin. The availability of plague alternative treatments is also crucial to improve the patient’s outcome and need to be implemented in the PNCP of the Malagasy Ministry of Health thus a new plague therapeutic scheme is currently under evaluation.

This situation has been considered to be an important alert and allowed the establishment of a contingency plan to counteract plague epidemics, particularly the occurrence of the pneumonic form in urban areas where the transmission risk at national and international levels is high.

## Abbreviations

APHAA: Anti-Plague Hospital of Ambohimiandra-Antananarivo
MDH: Miarinarivo District Hospital
MHA: Military Hospital of Antananarivo
PNCP: Plague National Control Program

## References

[1] Stenseth NC, Atshabar BB, Begon M, Belmain SR, Bertherat E, Carniel E (2008). Plague: past, present, and future. PLoS Med.

[2] Price PA, Jin J, Goldman WE (2012). Pulmonary infection by *Yersinia pestis* rapidly establishes a permissive environment for microbial proliferation. Proc Natl Acad Sci U S A.

[3] World Health Organization (2016). Plague around the world, 2010–2015. Wkly Epidemiol Rec.

[4] Brygoo ER (1966). Epidemiologie de la peste à Madagascar. Arch Inst Pasteur Madagascar.

[5] World Health Organization (2006). International meeting on preventing and controlling plague: the old calamity still has a future. Wkly Epidemiol Rec.

[6] Galimand M, Guiyoule A, Gerbaud G, Rasoamanana B, Chanteau S, Carniel E (1997). Multidrug resistance in *Yersinia pestis* mediated by a transferable plasmid. N Engl J Med.

[7] Khardori N, Elting L, Wong E, Schable B, Bodey GP (1990). Nosocomial infections due to *Xanthomonas maltophilia* (*Pseudomonas maltophilia*) in patients with cancer. Rev Infect Dis.

[8] Looney WJ, Narita M, Mühlemann K (2009). *Stenotrophomonas maltophilia*: an emerging opportunist human pathogen. Lancet Infect Dis.

[9] Sendrasoa FA, Razanakoto NH, Ranaivo IM, Andrianasolo RL, Randria MJDD, Antibiotic RRA (2016). Antimalarial Selfmedication in Antananarivo, Madagascar. Int J Infect Dis Ther.

[10] Chanteau S, Rahalison L, Ralafiarisoa L, Foulon J, Ratsitorahina M, Ratsifasoamanana L (2003). Development and testing of a rapid diagnostic test for bubonic and pneumonic plague. Lancet.

[11] Rasoamanana B, Rahalison L, Raharimanana C, Chanteau S (1996). Comparison of Yersinia CIN agar and mouse inoculation assay for the diagnosis of plague. Trans R Soc Trop Med Hyg.

[12] Rasoamanana B, Leroy F, Boisier P, Rasolomaharo M, Buchy P, Carniel E (1997). Field evaluation of an immunoglobulin G anti-F1 enzyme-linked immunosorbent assay for serodiagnosis of human plague in Madagascar. Clin Diagn Lab Immunol.

[13] Hinnebusch J, Schwan TG (1993). New method for plague surveillance using polymerase chain reaction to detect Yersinia pestis in fleas. J Clin Microbiol.

[14] Pechous RD, Sivaraman V, Stasulli NM, Goldman WE (2016). Pneumonic plague: the darker side of Yersinia pestis. Trends Microbiol.

[15] Butler TC, Greenough WB (1983). Plague and other Yersinia infections. Current topics in infectious disease.

[16] Perry RD, Fetherston JD (1997). *Yersinia pestis*--etiologic agent of plague. Clin Microbiol Rev.

[17] Bertherat E, Thullier P, Shako JC, England K, Koné ML, Arntzen L (2011). Lessons learned about pneumonic plague diagnosis from two outbreaks, Democratic Republic of the Congo. Emerg Infect Dis.

[18] Begier EM, Asiki G, Anywaine Z, Yockey B, Schriefer ME, Aleti P (2006). Pneumonic plague cluster, Uganda, 2004. Emerg Infect Dis.

[19] Ratsitorahina M, Chanteau S, Rahalison L, Ratsifasoamanana L, Boisier P (2000). Epidemiological and diagnostic aspects of the outbreak of pneumonic plague in Madagascar. Lancet.

[20] Nicodemo AC, Garcia Paez JI (2007). Antimicrobial therapy for *Stenotrophomonas maltophilia* infections. Eur J Clin Microbiol Infect Dis.

[21] Fujita J, Yamadori I, Xu G (1996). Clinical features of *Stenotrophomonas maltophilia* pneumonia in immunocompromised patients. Respir Med.

[22] Pathmanathan A, Waterer GW (2005). Significance of positive *Stenotrophomonas maltophilia* culture in acute respiratory tract infection. EurRespir J.

[23] Samonis G, Karageorgopoulos DE, Maraki S, Levis P, Dimopoulou D, Spernovasilis NA (2012). *Stenotrophomonas maltophilia* infections in a general hospital: patient characteristics, antimicrobial susceptibility, and treatment outcome. PLoS One.

[24] Apangu T, Griffith K, Abaru J, Candini G, Apio H, Okoth F, et al. Successful treatment of human plague with oral ciprofloxacin. Emerg Infect Dis. 2017;23(3):553–55. 10.3201/eid2303.161212 PMC5382724 28125398

[25] Heine HS, Hershfield J, Marchand C, Miller L, Halasohoris S, Purcell BK (2015). In vitro antibiotic susceptibilities of Yersinia pestis determined by broth microdilution following CLSI methods. Antimicrob Agents Chemother.

[26] Wong JD, Barash JR, Sandfort RF, Janda JM (2000). Susceptibilities of *Yersinia pestis* strains to 12 antimicrobial agents. Antimicrob Agents Chemother.

[27] Roberts L (2017). Echoes of Ebola as plague hits Madagascar. Science.

[28] Quan TJ, Tsuchiya KR, Carter LG (1979). Isolation of pathogens other than *Yersinia pestis* during plague investigations. J Wildl Dis.

[29] Bertherat E, Mueller MJ, Shako JC, Picardeau M (2014). Discovery of a leptospirosis cluster amidst a pneumonic plague outbreak in a miners’ camp in the Democratic Republic of the Congo. Int J Environ Res Public Health.

